# Correlation analysis of *IL-11* polymorphisms and Hirschsprung disease subtype susceptibility in Southern Chinese Children

**DOI:** 10.1186/s12920-020-00867-x

**Published:** 2021-01-19

**Authors:** Hong Zhang, Jing-Lu Zhao, Yi Zheng, Xiao-Li Xie, Li-Hua Huang, Le Li, Yun Zhu, Li-Feng Lu, Tu-Qun Hu, Wei Zhong, Qiu-Ming He

**Affiliations:** Department of Pediatric Surgery, Guangzhou Institute of Pediatrics, Guangzhou Women and Children’s Medical Center, Guangzhou Medical University, 9 Jinsui Road, Guangzhou, 510623 Guangdong China

**Keywords:** Hirschsprung's disease, *IL-11*, Polymorphisms, Hirschsprung's disease-associated enterocolitis

## Abstract

**Background:**

Hirschsprung disease (HSCR) is a hereditary defect, which is characterized by the absence of enteric ganglia and is frequently concurrent with Hirschsprung-associated enterocolitis (HAEC). However, the pathogenesis for HSCR is complicated and remains unclear. Recent studies have shown that pro-inflammatory cytokines such as interleukin-11 (*IL-11*) are involved in the enteric nervous system's progress. It was found that IL-11 SNPs (rs8104023 and rs4252546) are associated with HSCR in the Korean population waiting for replication in an independent cohort. This study evaluated the relationship between *IL-11* and the susceptibility of patients to HSCR by performing subphenotype interaction examination, HAEC pre-/post-surgical patient-only association analysis, and independence testing.

**Methods:**

In this study, a cohort consisting of children from Southern China, comprising 1470 cases and 1473 controls, was chosen to examine the relationship between two polymorphisms (rs8104023 and rs4252546 in *IL-11*) and susceptibility to HSCR by replication research, subphenotype association analysis, and independence testing.

**Results:**

The results showed that *IL-11* gene polymorphisms (rs8104023 and rs4252546) are not associated with the risk of HSCR in the Chinese population. The results of both short-segment and long-segment (S-HSCR and L-HSCR) surgery (3.34 ≤ OR ≤ 4.05, 0.02 ≤ P ≤ 0.04) showed that single nucleotide polymorphisms (SNP) rs8104023 is associated with susceptibility to HAEC.

**Conclusions:**

This study explored the relationship between genetic polymorphisms and susceptibility to HAEC in HSCR subtypes for the first time. These findings should be replicated in a larger and multicentre study.

## Background

Hirschsprung disease (HSCR) is a congenital disability characterized by the complete or partial absence of intestinal ganglia [1]. A significant racial difference in the HSCR incidence has been observed, and it is mostly found in Asia (28 per 100,000 live births) [2]. In addition, males are frequently affected at a ratio of 4:1 [3]. Patients are were classified into short-segment HSCR (S-HSCR), long-segment HSCR(L-HSCR), and total colonic aganglionosis(TCA) [4]. Intestinal obstruction or chronic constipation remains the primary clinical manifestation of HSCR. HSCR is frequently concurrent with Hirschsprung-associated enterocolitis (HAEC) and is sometimes becomes a life-threatening complication [5, 6].

RET proto-oncogene encoding receptor tyrosine kinases can only explain 50% of patients with genetic HSCR and 15–20% with sporadic HSCR [7]. Other markers can only explain less than 5% of the total HSCR incidence of HSCR [1–3]. This thus indicates that there may be other chromosomal abnormalities that may be responsible for the development of HSCR. Recent studies show that pro-inflammatory cytokines, such as *IL-11*, participate in neurite growth, and myenteric neurons, and other functions of ENS. For instance, *IL-11* plays an important role in the survival, maturation, and myelin formation of oligodendrocytes. A study by Tomuschat et al*.* reported that the pro-inflammatory cytokines were observed in both the aganglionic and ganglionic and aganglionic regions of the bowels of patients with HSCR [8, 9]. A population-based genome-wide association study (GWAS) on the Korean population was performed by Kim et al*.,* showing that a number of SNPs on *IL-11* such as rs1126760, rs4252546, and rs8104023 have statistically significant associations with HSCR based on 187 cases and 283 controls [10, 11]. In another study on the German population, the over-expression of > 7GT repeat-subtypes was observed in 103 HSCR cases during the extension of GT dinucleotide repeat sequences in the enhancer/promoter region of *IL-11* [12]. A variety of studies have been carried out on additional cases of different sizes, ethnic backgrounds, and nationalities. As a result, a GWAS on the Korean population showed that single nucleotide polymorphisms (SNPs), including rs1126760, rs4252546, and rs8104023, are associated with HSCR. In addition, the incidence of HSCR varies from race to race. Therefore, it is necessary to require further validation in another cohort. The association between *IL-11* gene polymorphisms and HSCR susceptibility was investigated by conducting a case–control study of two previously confirmed SNPs (rs8104023 and rs4252546) related to the pathogenesis of HSCR based on an independent Chinese sample (1470 cases and 1473 controls). However, the association of selected SNPs with HSCR failed to be further verified. In addition, rs8104023 was found to be a relevant SNP, whose relationship with HAEC susceptibility was examined.

## Methods

### Characteristics of study subjects

DNA samples of 2943 participants (1470 HSCR patients and 1473 controls) were selected from Guangzhou Women and Children's Medical Center (Additional file 1: Supplementary Table 1). This table summarizes the subclinical information on a South Chinese population consisting of 1470 HSCR patients and 1473 healthy controls. Age and gender distributions were similar across cases and controls (*P* > 0.05). These participants were diagnosed with HSCR as per the histological testing of enteric aganglionosis (using biopsy specimens). Patients with HSCR were divided into three subgroups, including 1033 S-HSCR (70.27%), 294 L-HSCR (20.00%), and 82 TCA (5.58%). In this study, the prevalence of L-HSCR and TCA patients exceeded the general prevalence of HSCR subgroups (70–80% of S-HSCR, 15–20% of L-HSCR, and 5% of TCA). Besides, the blood samples of 1473 unaffected controls were obtained from a cohort group. In addition, all the controls were collected without a history of HSCR and neurological disease. Written informed consent was obtained from all the participants' guardians, and the study protocol was approved by the Institutional Review Board of the hospital.

**Table 1:** Patient-only associations of *IL-11* SNPs with HSCR Enteritis stratification

SNP	A1/A2	E_before operation_SHCSR				E_before operation_LHCSR				E_before operation_TCA			
		F_A	F_U	P	OR	F_A	F_U	P	OR	F_A	F_U	P	OR
rs8104023	C/T	0.02	0.01	**0.02**	4.05 (1.18–13.93)	0	0.03	**0.03**	NA	0	0	NA	NA
rs4252546	T/C	0.26	0.27	0.71	0.94 (0.70–1.28)	0.26	0.23	0.57	1.17 (0.68 ~ 2.02)	0.20	0.17	0.68	1.29 (0.38 ~ 4.30)

		E_after operation_SHCSR				E_after operation_LHCSR				E_after operation_TCA			
rs8104023	C/T	0.02	0.01	**0.04**	3.34 (1.01–11.04)	0.00	0.03	**0.03**	NA	0.00	0.00	NA	NA
rs4252546	T/C	0.25	0.27	0.49	0.90 (0.66–1.23)	0.22	0.28	0.25	0.73 (0.43–1.25)	0.18	0.31	0.19	0.49 (0.17–1.44)

SNP, Single Nucleotide Polymorphism; A1/A2 indicates the risk-allele and protective allele to disease; F_A/F_U indicates risk-allele frequency of the SNP in cases or controls; SHSCR, short-segment HSCR; L-HSCR, long-segment HSCR; TCA, total colon aganglionosis; The P-value indicates the significance based on allelic association tests; The calculation of odds ratio (OR) is also based on the risk-allele of each SNP

### Single nucleotide polymorphism genotyping and selection for the replication study

A previous GWAS study of the Asian population described the associations of *IL-11* SNPs (rs1126760, rs4252546, rs8104023) with HSCR [11]. The above-mentioned SNPs were replicated in Southern Chinese Children to reconfirm the relationship between *IL-11* and HSCR. Three SNPs were chosen according to 1) 1 SNP exceeded the significance of genome-wide association (P < 1 × 10^–8^). 2) 2 SNPs showed limited linkage disequilibrium (LD) (r^2^ < 0.5). The significant signals in potential loci were selected based on the minor allele frequency (< 5%) in the Chinese population. Quality control of SNPs was conducted as follows: (1) 2 SNPs exceeded the filtering criteria with a missing rate of less than 10% (1 SNP was removed). 2) Subjects with a 10% missing rate were excluded. Further analysis of 1469 cases and 1473 controls after the quality control included two SNPs (rs8104023 and rs4252546).

### Subphenotype stratification and association analysis

The allelic test was used to compare the risk-allele frequency, and PLINK 1.9 was used to analyze additional tests, including the Cochran-Armitage trend test, genotype test in 3 × 2 contingency tables, dominant and recessive model test, logistic regression, the SNPs were explored for the association with HSCR. Moreover, a comparison of controls and cases with a certain subphenotype was analyzed using subphenotype stratification.

### Independence testing

This study used HaploView was used to obtain linkage disequilibrium patterns and values [13]. SNPTEST v2.5b was used to perform logistic regression tests, and the independent contribution of SNPs to the association with HSCR in a single locus was analyzed, thereby adjusting the effect of a specific SNP in the same locus.

## Results

### Associations of IL-11 SNPs with HSCR

The replication study used two SNPs (rs8104023, rs4252546) for the association analysis to identify multiple independent variants related to HSCR. Genotype distribution of all the SNPs failed to violate the Hardy–Weinberg Equilibrium (HWE) controls (P = 0.47). The association of SNPs cannot be replicated, which was inconsistent with the report by Kim et al*.* [11]. The physical diagram of *IL-11* shows detailed locations of SNPs. Based on gender, HAEC, constipation, and subtypes, stratification analysis was done to assess the association of the two selected polymorphisms with the risk of HSCR. To explore the effect of polymorphisms, the relationship between *IL-11* polymorphisms and risks of HSCR subgroups (S-HSCR, L-HSCR, TCA) was also analyzed based on the aganglionic segment range of HSCR. Interestingly, both S-HSCR and L-HSCR subgroups showed a significant increase in signals, which showed a significant association of rs8104023 with the enterocolitis risk no matter before or after the operation. However, *IL-11* SNPs (rs8104023, rs4252546) were not significantly associated with HSCR. In the analysis of constipation, no significant association with HSCR, even in the comparison of HSCR subgroups, was observed.

### Stratification analysis of IL-11 gene polymorphisms with the complication of HSCR susceptibility

The most common and serious complication of HSCR is Hirschsprung-associated enterocolitis (HAEC), which happens either before or after the operation. Therefore, the relationship between two *IL-11* SNPs and HAEC was analyzed, and the relationship between the three selected SNPs and pre-/post-operative HAEC was investigated by patient-only analysis (Table 1). An association between SNP rs8104023 and the susceptibility to HAEC before and after surgery in S-HSCR (OR = 4.05, P = 0.02 in before operation; OR = 3.34, P = 0.04 in after operation) and L-HSCR (P = 0.03 in before operation; P = 0.03 in after operation) was observed.

### Independence testing of replicated SNPs in IL-11

**Fig. 1:**
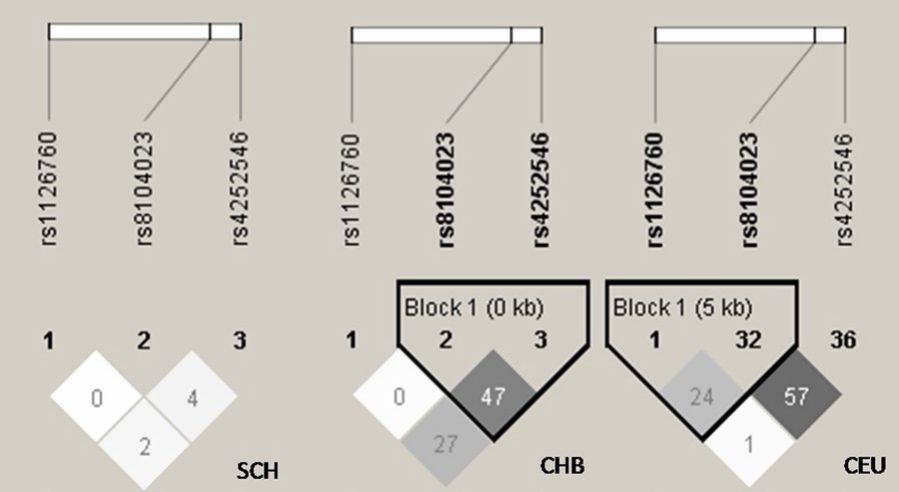
The linkage disequilibrium patterns (LD) of the SNPs in *IL-11.* SCH, Singapore Chinese. CHB, Han Chinese in Beijing, China. CEU, Residents from Utah with ancestry from northern and western Europe. LD r^2^ prime charts from HaploView that summarize the LD patterns in SCH, CHB, and CEU are shown. The numbers in the boxes are the pairwise correlation coefficient r^2^ between respective SNPs. Darker shades of gray indicate a higher value of LD. Lighter shades of gray represent a lower value of LD. Rs1126760 was not included in the replication experiment. Rs8104023 and rs4252546 cluster together because of physical proximity

Figure 1 shows the LD patterns of replicated SNPs on the basis of varied populations from HapMap data like Chinese Singaporean, Han Chinese, and Utah residents with ancestors from Northern and Western Europe. SNP rs1126760 was included for analysis due to its association with HSCR, according to report (11). SNP rs1126760 showed limited LD compared with other SNPs in Utah residents with ancestors from Western and Northern Europe (Fig. 1, r^2^ = 0.24). SNPs rs8104023 and rs4252546 showed medium to high LD in Chinese populations (r^2^ = 0.47).

## Discussion

HSCR results from many genetic factors. Various studies have shown that candidate gene, including SOX10 and Glial cell line-Derived Neurotrophic Factor (GDNF), are important for the diagnosis of HSCR. Several researches have shown the etiology of HSCR. However, the precise mechanism remains unknown. This study showed that two SNPs (rs8104023, rs4252546) in the *IL-11* gene were unrelated to the risk of HSCR in Chinese populations. Furthermore, *IL-11* gene polymorphisms may play a role in HSCR-associated enterocolitis.

*IL-11* belongs to the *IL-6* cytokine family and can stimulate megakaryocytopoiesis, which is essential in pro-inflammatory functionality [14]. *IL-11* has been reported to affect the progress of the enteric nervous system [15]. The fluctuation in IL11 protein levels influences neural maturation and inflammation that is often seen as a characteristic symptom of HSCR. In the study of Kim et al*.*, nine SNPs of the IL11 gene were selected, and several SNPs were identified to have a statistical and significant association with HSCR. The frequencies of rs4252546 and rs8104023 were higher in HSCR cases than those in unaffected subjects [11]. In contrast, the study by Haase et al*.* showed that these two SNPs in the IL11 gene were not significantly related to HSCR of the German population (128 healthy controls and 103 HSCR patients). This study further explored whether the same situation would be observed in a larger Chinese population. In this study, SNP rs4252546 is a putative binding site of NF-1 and may affect gene expression (pcorr = 9.70 9 10_4 in combined analysis) [10]. Besides, rs4252546 and rs8104023 exist in a potential regulatory region and an intronic region, respectively. These findings reveal that the *IL-11* gene may interact with other genes to exert a limited role in regulating and controlling the aganglionic segment during the ENS development.

However, no study has explored the relationship between *IL-11* gene polymorphisms and susceptibility to HSCR in a Southern Chinese population. This study found no association between the 2 SNPs with HSCR. However, small sample size may negatively affect ethnic diversity or sample size, possibly reflected by different genetic backgrounds. The independence test (Fig. 1) shows that rs1126760 exhibited limited LD with all other SNPs in the CEU. Rs8104023 and rs4252546 exhibited moderate to high LD between each other in the CHB.

Our results showed no significant association with HSCR regarding enterocolitis. Similarly, research by Liao et al. provided evidence that *IL-11* rs8104023 is not significantly associated with the risk of gastric cancer based on 880 Chinese cases [16]. A study by Haase et al*.* showed that *IL-11* rs4252546 has no significant associations with HSCR based on a German population (103 cases and 128 controls) [12]. This might be due to limited samples. Therefore, the patients were categorized into different groups based on clinical HSCR subtypes. This methodology showed an increased association of rs8104023 with S-HSCR and L-HSCR both before and after surgery. HEAC is a well-known complication of HSCR that occurs at any stage and may potentially cause death. There are numerous theories explaining the occurrence of HEAC, but the reasons remain unclear. According to the study by Greenwood-Van Meerveld et al*.*, *IL-11* restores the ability of longitudinal muscles, thus reversing the inflammatory response and producing active tension in both the jejunum and colon [17]. This demonstrates that the occurrence of HSCR-associated enterocolitis may be reduced by *IL-11* polymorphisms. *IL-11* promoter variants may modulate the expression of *IL-11* to increase the susceptibility to HEAC, thereby leading to abnormal neuronal maturation and colonic inflammation. However, the functional mechanism of *IL-11* and HEAC is unclear, thereby requiring further studies with larger sample sizes, different populations, and functional evaluations to verify these results.

## Conclusions

Based on other studies, this study uses additional *IL-11* SNPs for fine mapping and replication and then investigates associations. Both *IL-11* gene polymorphisms (rs8104023 and rs4252546) may be irrelevant to the risk of HSCR in the Chinese population but play a role in HAEC, which may provide a new way to study HEAC. To address these possibilities, further studies with larger sample sizes, different populations, and functional evaluations should be carried out.

## Supplementary information

**Additional file 1**. The subclinical information collected for the subjects in this study.

## Data Availability

The datasets generated and analyzed during the current study are available from the corresponding author on reasonable request.

## Abbreviations

SNP: single nucleotide polymorphism
CHR: chromosome
BP: base pair of where the SNP is located
A1/A2: indicates the risk-allele and protective allele to disease
F_A/F_U: indicates the risk-allele frequency of the SNP in cases or controls
The P-value: indicates the significance based on allelic association tests
The calculation of the odds ratio (OR): is also based on the risk-allele of each SNP
CI: confidence interval
HSCR: Hirschsprung disease
*IL-11*: interleukins-1
HAEC: Hirschsprung's disease-associated enterocolitis
ENS: the enteric nervous system
GWAS: genome-wide association study
S-HSCR: short-segment HSCR
L-HSCR: long-segment HSCR
TCA: total colonic aganglionosis
