# Supplementary material for: Correlation analysis of IL-11 polymorphisms and Hirschsprung disease subtype susceptibility in Southern Chinese Children
Source: BMC Med Genomics. 2021 Jan 19;14:21. doi: 10.1186/s12920-020-00867-x (PMC7814452; doi:10.1186/s12920-020-00867-x)
Supplement: Supplementary file 1 — Additional file 1. The subclinical information collected for the subjects in this study. [file 12920_2020_867_MOESM1_ESM.docx]

**Supplementary Table 1**. The subclinical information collected for the subjects in this study

| HSCR subphenotype | Cases (n=1470) | % | Controls (n=1473) | % |
| --- | --- | --- | --- | --- |
| Subjects |  |  |  |  |
| Age range (Months) | 8.37±20.50 |  | 18.61±19.75 |  |
| ≤2 | 725 | 49.32% | 458 | 31.09% |
| >2 | 745 | 50.68% | 1015 | 68.91% |
| Gender |  |  |  |  |
| Females | 240 | 16.33% | 967 | 65.65% |
| Males | 1230 | 83.67% | 506 | 34.35% |
| Clinical manifestation |  |  |  |  |
| SHCSR | 1033 | 70.27% |  |  |
| LHCSR | 294 | 20.00% |  |  |
| TCA | 82 | 5.58% |  |  |
| Total intestine | 3 | 0.20% |  |  |
| Enteritis_before_operation | 261 | 17.76% |  |  |
| Enteritis_after_operation | 249 | 16.94% |  |  |

**Supplementary Table 2.** Primer sequences used for detecting the two SNPs in *IL-11*

| WELL | SNP | 1st-PCRP | 2nd-PCRP | EXT1_SEQ | EXT2_SEQ |
| --- | --- | --- | --- | --- | --- |
| W2 | rs4252546 | ACGTTGGATGCCTCTGATCCTCTTTGCTTC | ACGTTGGATGAAGGACAGACTGAGACAGAG | AGACAGAGACAGGGAGAC | AGACAGAGACAGGGAGAT |
| W1 | rs8104023 | ACGTTGGATGAATCCCAGGGAGTCTCCCG | ACGTTGGATGAGGGACGCAGGGACTGGTG | tatgtAGGTGACCCCCATCGA | tatgtAGGTGACCCCCATCGG |
